# Supplementary material for: The Thermodynamic Fingerprints of Ultra-Tight Nanobody–Antigen Binding Probed via Two-Color Single-Molecule Coincidence Detection
Source: Int J Mol Sci. 2023 Nov 15;24(22):16379. doi: 10.3390/ijms242216379 (PMC10671529; doi:10.3390/ijms242216379)
Supplement: Supplementary file 1 [file ijms-24-16379-s001.zip › ijms-2685075-supplementary.pdf]

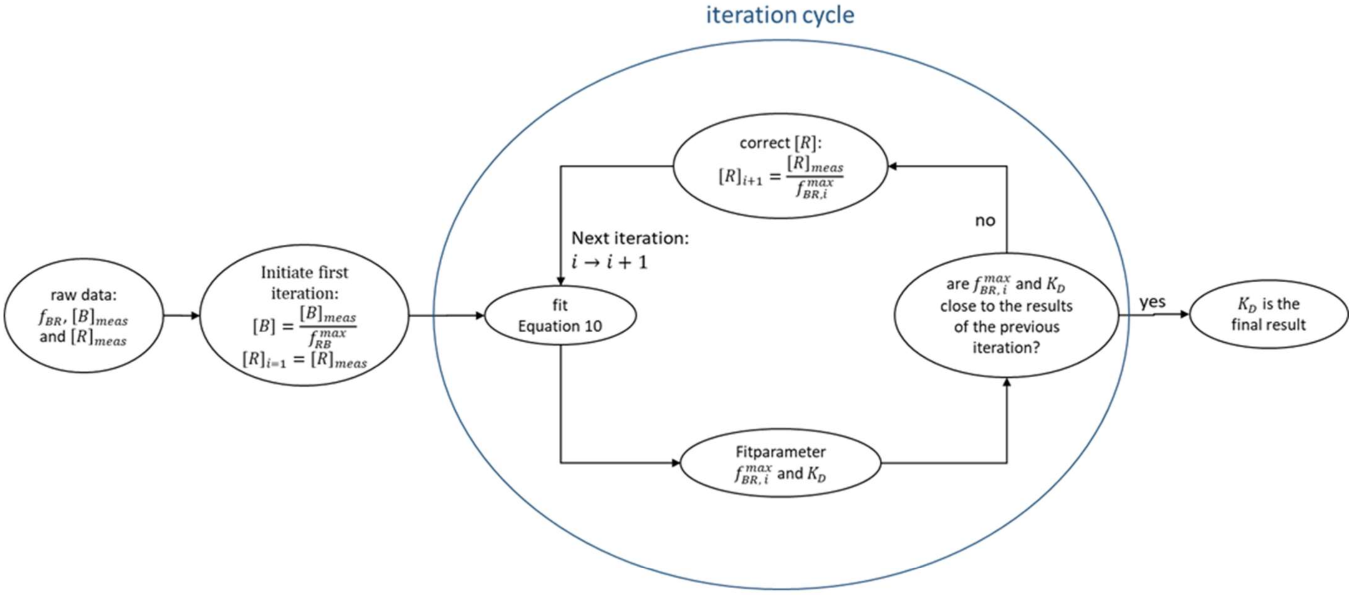


$$f_{BR} = f_{BR\ i}^{max} \frac{([B]+[R]_i+K_D) - \sqrt{([B]+[R]_i+K_D)^2 - 4[B]\cdot[R]_i}}{2[B]}$$

$$[R]_{i+1} = \frac{[R]}{f_{BR\ i}^{max}} \qquad [B] = \frac{[B]_{meas}}{f_{RB}^{max}}$$

**Scheme S1:** Iterative correction of the quadratic model: In this scheme the parameter *i* gives the number of the iteration step. In the first iteration step ($i$=1) the uncorrected data of $[R]$ ($=[R]_{i=1}$), $f_{BR}$ and the corrected data for $[B]$ (see equation above) are fitted using the equation shown above to obtain the fit parameters $f_{BR}{}^{max}{}_{i=1}$ and $K_D$. For the second iteration step the red concentration is corrected using $f_{BR}{}^{max}{}_{i=1}$ to obtain $[R]_{i=2}$. Now the equation is used again to fit the data with $[R]_{i=2}$. The resulting values of $f_{BR}{}^{max}{}_{i=2}$ and $K_D$ differ from the ones of the first iteration. For the next iteration the red concentration is corrected using $f_{BR}{}^{max}{}_{i=2}$ to obtain $[R]_{i=3}$. The procedure is repeated until the resulting values of $f_{BR}{}^{max}{}_i$ and $K_D$ do not change anymore.

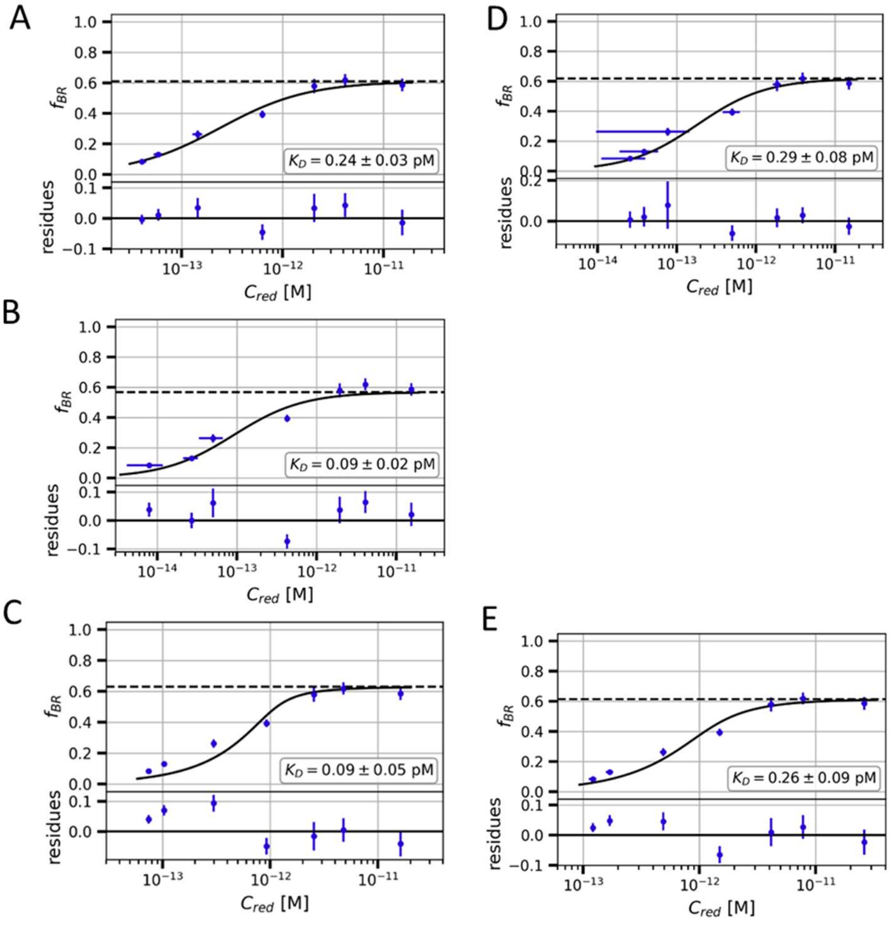


**Figure S1:** Detailed analysis of DNA hybridization binding reaction. The measurements were performed with $[B]$ ~ 1.5 pM. Fits with uncorrected data points are shown in panels on the left side (A-C) and with corrected $[R]_{\mathrm{free}}$ and $K_D$ values on the right (panels D,E). In A and D hyperbolic fits are shown for $[R]_{\mathrm{free}}$ calculated with $f_{RB}$, while in panel B a hyperbolic fit was performed with $[R]_{\mathrm{free}}$ calculated by using $f_{BR}$. In the latter case, corrected data were not available. In panels C and E quadratic fits were performed. In all graphs the dashed lines represent the $f_{BR}^{\mathrm{max}}$ values as obtained in each fit.

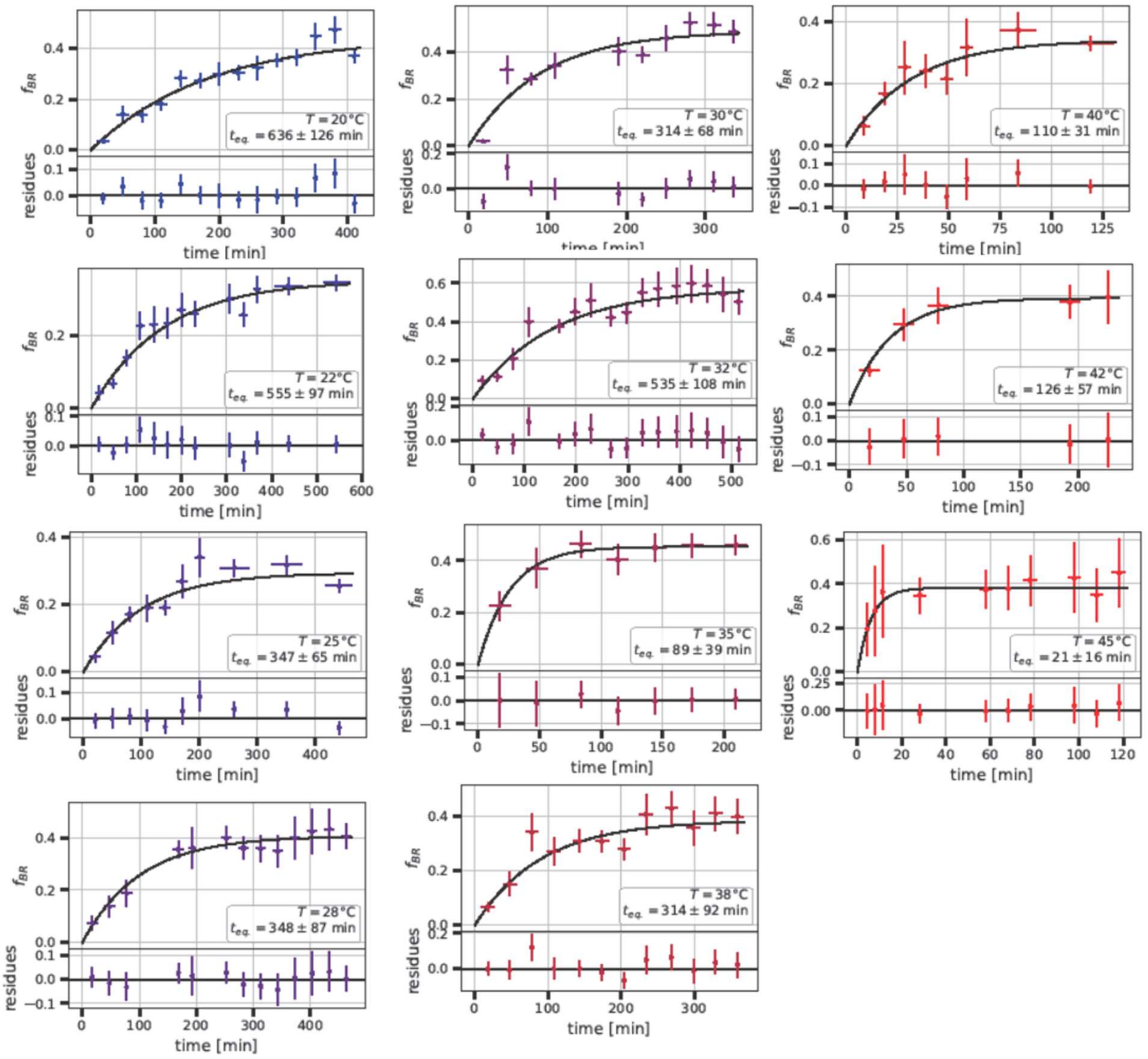


**Figure S2:** Nanobody-EGFP binding interaction. Equilibrium time curves from which the fit gives equilibrium times $t_{eq}$= $5 \cdot t_{1/2}$ (see Figure 1) as obtained for measurements at different temperatures.

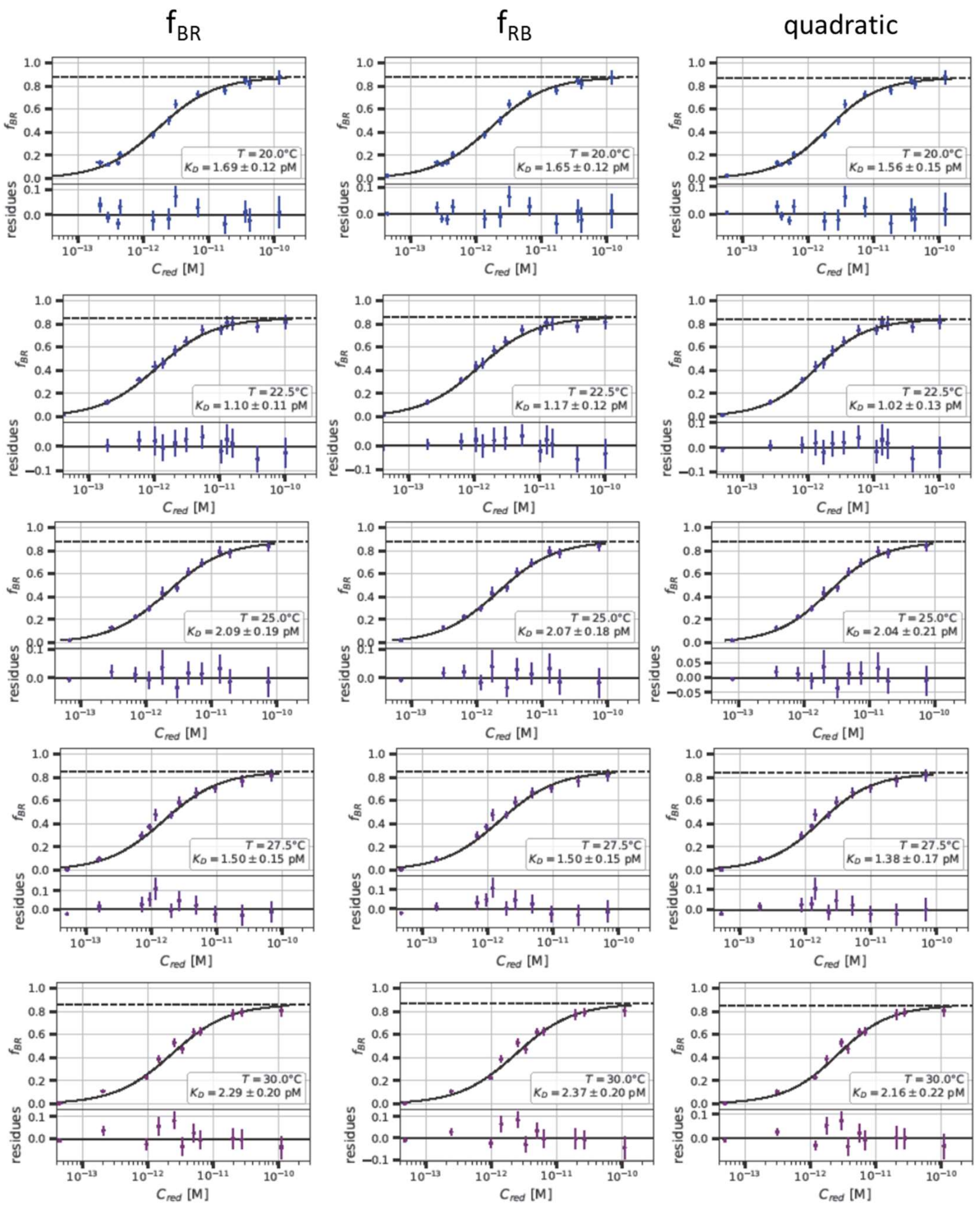


**Figure S3:** Nanobody-EGFP binding interaction. Hyperbolic fits are shown for uncorrected $[R]_{\text{free}}$ and uncorrected $K_D$ values which were calculated using $f_{RB}$ and calculated using $f_{BR}$ as well as quadratic fits with uncorrected data. In all graphs the dashed lines represent the $f_{BR}^{\max}$ values as obtained in each fit. In contrast to results obtained from the DNA sample (Figure S1), we got extremely well matching $K_D$ values here. The deviation between results from both hyperbolic fits and the quadratic fit are rather small. Here the data for measurements between 20 ° and 30 °C is shown.

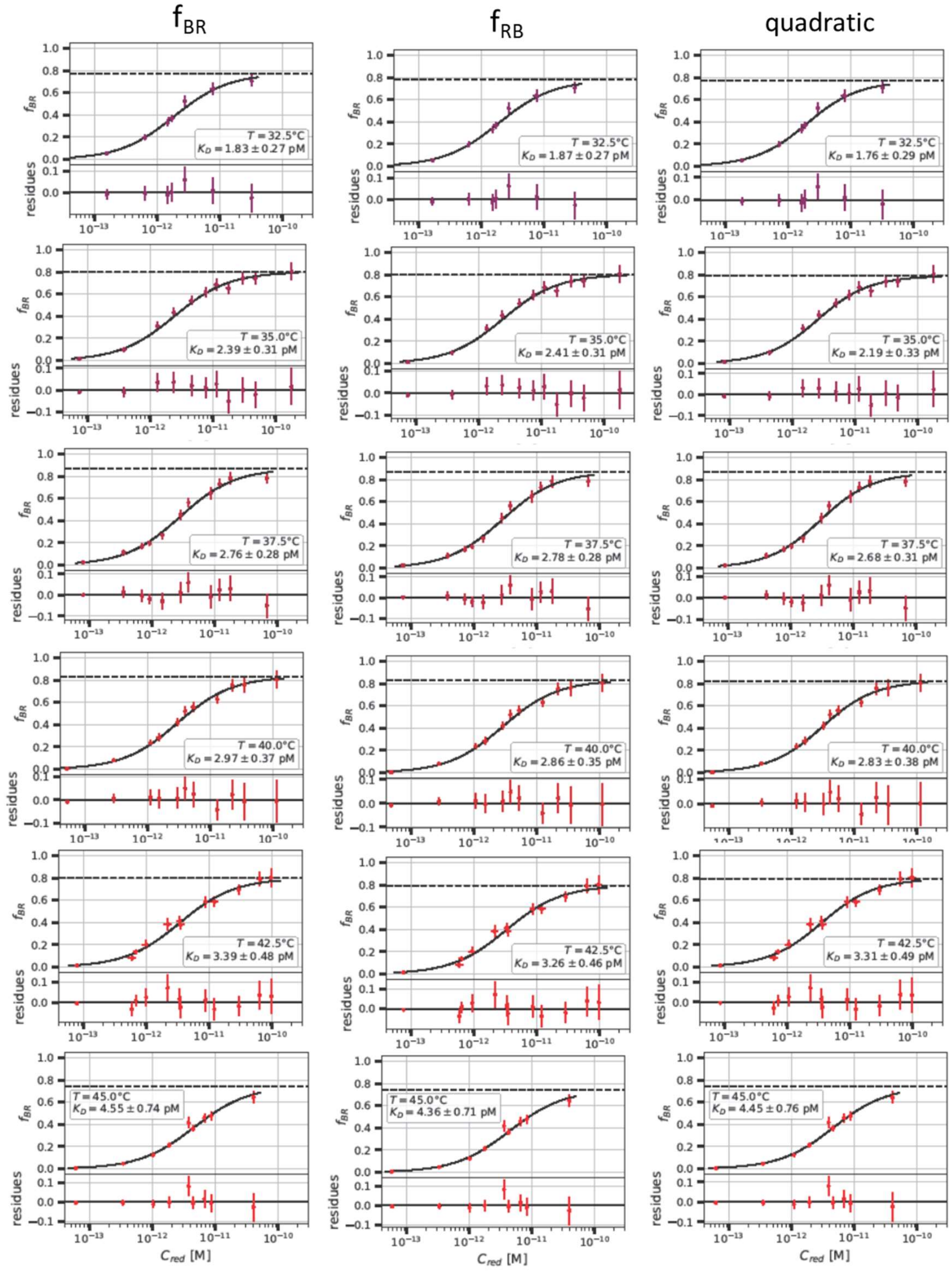


**Cont. Figure S3:** Continuation for temperatures between 32.5 and 45 °C.

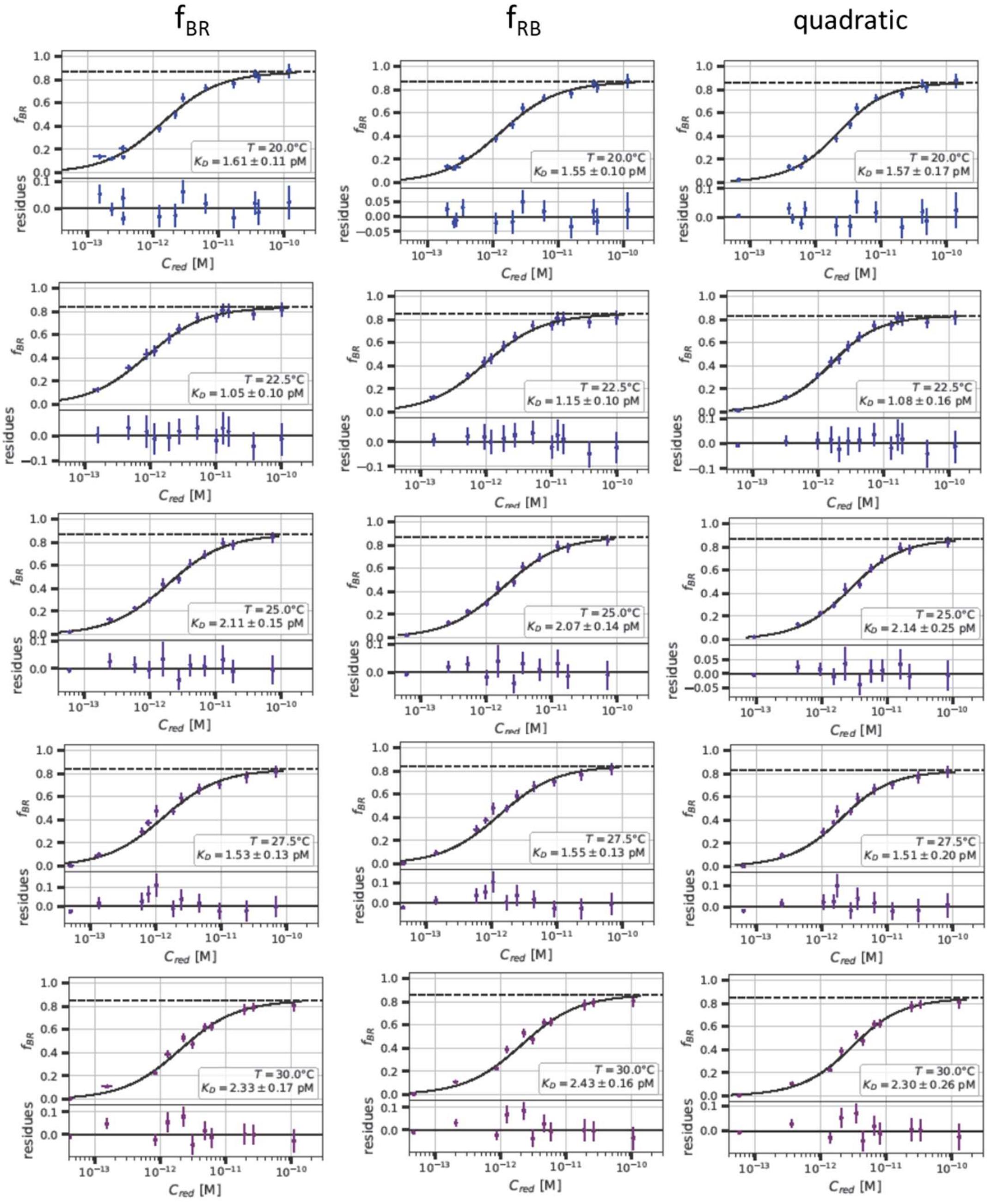


**Figure S4:** Nanobody-EGFP binding interaction. Hyperbolic fits are shown for corrected $[R]_{\text{free}}$ and uncorrected $K_D$ values which were calculated using $f_{RB}$ and calculated using $f_{BR}$ as well as quadratic fits with corrected data. In all graphs the dashed lines represent the $f_{BR}^{\max}$ values as obtained in each fit. Again, also for the corrected data, we got extremely well matching $K_D$ values here. The deviation between results from both hyperbolic fits and the quadratic fit are rather small. Here for measurements between 20 ° and 30 °C.

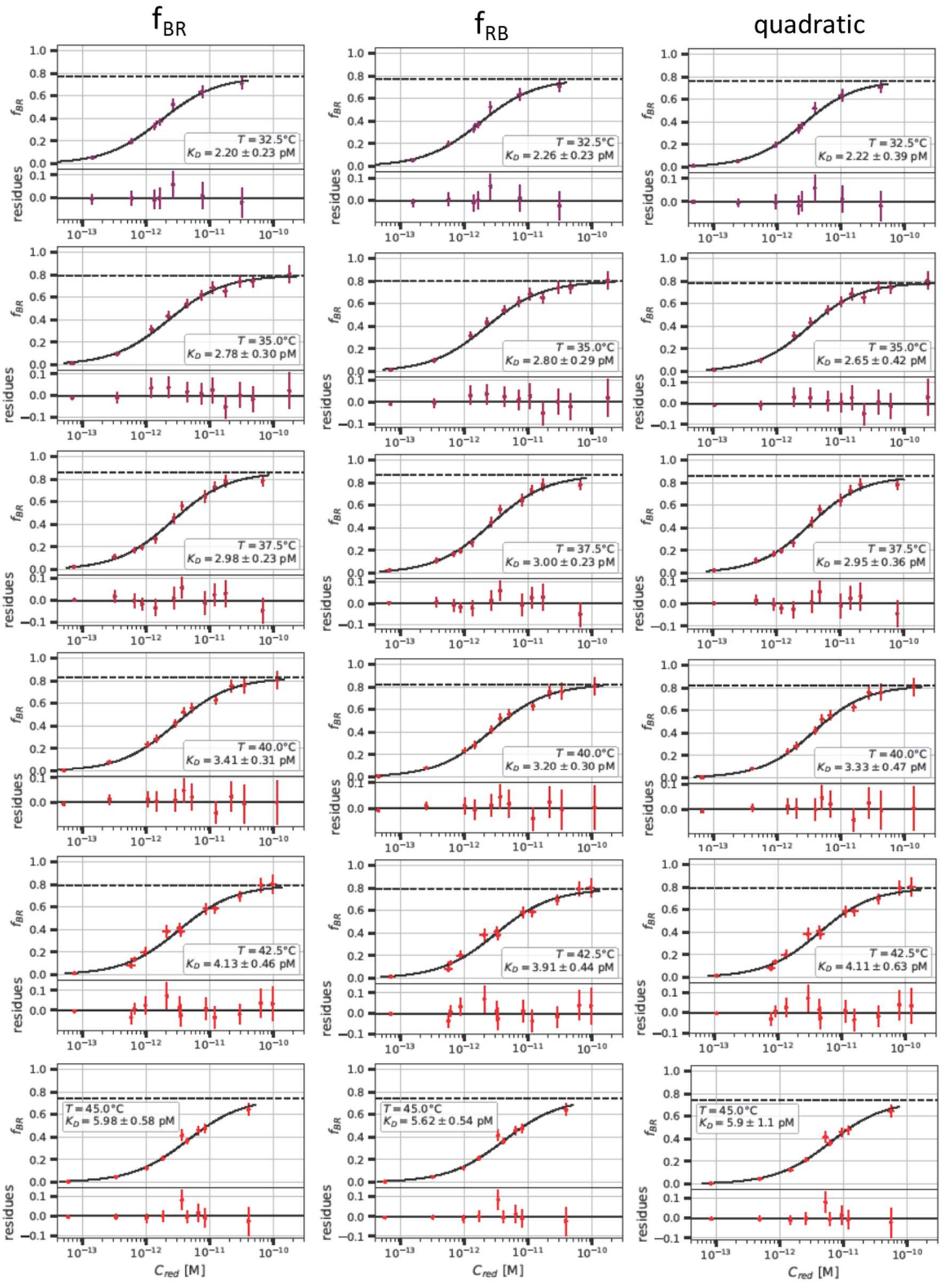


**Cont. Figure S4:** Continuation for temperatures between 32.5 and 45 °C.

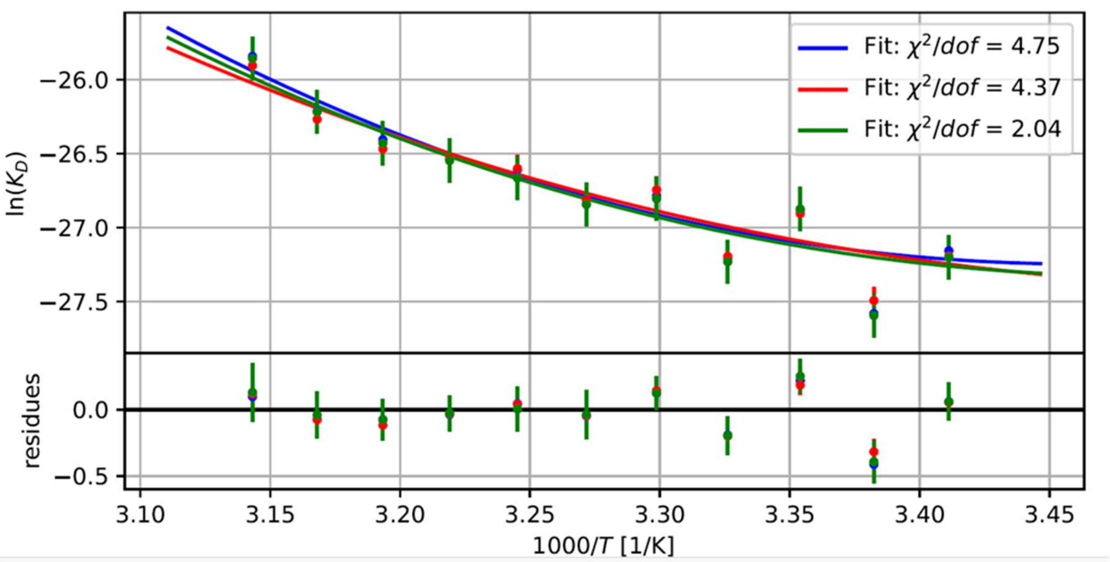


| model | hyp. $f_{BR}$ (blue) | hyp. $f_{RB}$ (red) | quadr. (green) |
|---|---|---|---|
| ΔG [kJ/mol] | -67.181 ± 0.083 | -67.152 ± 0.079 | -67.24 ± 0.13 |
| ΔH [kJ/mol] | -22.4 ± 5.3 | -26.6 ± 5.2 | -24.7 ± 8.6 |
| ΔS [kJ/(mol K)] | 0.150 ± 0.018 | 0.136 ± 0.017 | 0.143 ± 0.029 |
| T·ΔS [kJ/mol] | 44.8 ± 5.3 | 40.5 ± 5.2 | 42.5 ± 8.6 |
| $\Delta C_p$ [kJ/(mol K)] | -2.39 ± 0.72 | -1.58 ± 0.70 | -2.1 ±1.2 |

**Figure S5:** Nanobody-EGFP binding interaction. Van't Hoff plots with $K_D$ values as obtained from hyperbolic and quadratic fits by using corrected data (see Figure S4). In the lower table the corresponding thermodynamic parameters are given.
